# The effect of web-based education on self-care behaviors in cardiovascular patients: application of the pender’s health promotion model

**DOI:** 10.1186/s13690-024-01299-0

**Published:** 2024-05-09

**Authors:** Homamodin Javadzade, Hakime Vahedparast, Azime Khodaminasab, Rahim Tahmasebi, Mahnoush Reisi, Jamile Kiani

**Affiliations:** 1 Department of Health Education and Health Promotion, Bushehr University of Medical Sciences, Bushehr, Iran; 2 Department of Medical-Surgical Nursing, School of Nursing and Midwifery, Bushehr University of Medical Sciences, Bushehr, Iran; 3 Department of Epidemiology & Biostatistics, School of Health, Bushehr University of Medical Sciences, Bushehr, Iran; 4 Clinical Research Development Center, Shohadaye-Khalije-Fars Hospital, Bushehr University of Medical Sciences, Bushehr, Iran

**Keywords:** Angioplasty, Cardiovascular disease, Health promotion, Self-care, Web-based

## Abstract

**Background:**

Coronary artery disease is the most common cardiovascular disease, the mortality rate of which is increasing significantly. The most important way to prevent a second attack in patients undergoing angioplasty is self-care, which can be influenced by several factors such as the patient’s beliefs. Thus, the present study aimed to determine the effect of a web-based intervention based on the Pender’s health promotion model in patients with cardiovascular disease.

**Methods:**

The present clinical trial study was conducted with 99 patients undergoing angioplasty treatment referring to Bushehr Heart Specialist Center. Random sampling was done and the participants were divided into two groups (50 subjects in intervention group and 49 subjects in control group). The data collection tool was a three-part questionnaire (including demographic information, a researcher-made questionnaire based on the health promotion model constructs, and self-care behaviors), which was completed in three stages (before, two weeks, and three months after the intervention). In addition to routine hospital services, the intervention group received multimedia training based on the constructs of the Pender’s health promotion model from the website. The control group received usual hospital services. Data were analyzed with chi-square, independent T-test and repeated measure ANOVA using SPSS-22 software.

**Results:**

The results showed that 2 weeks and 3 months after the intervention, the mean scores of perceived benefits, perceived self-efficacy and perceived social support had a significant increase in the intervention group compared to the control group, but the mean score of perceived barriers had a significant decrease in the intervention group (*p* < 0.001). Regarding self-care behaviors, after the intervention, the mean scores of self-care behaviors, physical activity, healthy diet, medication adherence and stress management had a significant increase in the intervention group compared to the control group (*p* < 0.001), but no significant was observed between the two groups in terms of changes in the non-smoking (*p* = 0.38).

**Conclusion:**

The results of the study showed that the web-based educational intervention based on the health promotion model is useful in improving the self-care behaviors of cardiac patients undergoing angioplasty. Nursing education and care have a great role in improving the self-care behaviors of cardiovascular patients.

**Trial registration:**

Registration number: IRCT2017080635429N2. Registration date: 09/03/2017 (https://en.irct.ir/trial/26775).

**Table:** 

**Text box 1. Contributions to the literature**
• Web-based Education based on Pender’s health promotion model leads to improved self-care in patients with cardiovascular diseases.
• Web-based education provides patients with the ability to store information, add new information to previous materials, reproducibility of educational materials, and access to information at any time. This can make patients maintain their self-care and be very effective in achieving rehabilitation and secondary preventive services.
• The educational managers of cardiac treatment-educational centers can use web-based education in their educational planning for the continuity of care and more interaction of patients with service providers after discharge.

## Introduction

Cardiovascular disease is the first cause of mortality and disability worldwide, according to the World Health Organization [1]. The report of the World Health Organization in 2020 shows that cardiovascular diseases, as one of the non-communicable diseases, are the most important cause of death across the world [2]. In Iran, as a developing country with low-income level, 26.4% of recorded deaths were due to cardiovascular diseases [3]. Coronary interventions through the skin, including the angioplasty technique, have played a significant role over the past 30 years in the prevention, treatment, and rehabilitation of cardiovascular diseases. Because of less cost and risk of angioplasty compared to open heart surgery, today many patients worldwide are treated with this technique. Coronary angioplasty can help reduce or eliminate the symptoms and improve the patient’s condition by opening narrowed vessels [4].

Patients undergoing coronary angioplasty need long-term self-care and lifestyle modifications. Self-care entails an individual’s learned, conscious and purposeful actions and behaviors to ensure, maintain and improve health [5]. The American Heart Association has proposed self-care behaviors such as smoking cessation, regular hospital visits, medication adherence, diet management, adequate physical activity, and weight control to prevent a second attack or another adverse cardiac event in patients undergoing angioplasty [6].

In most cases, patients treated with angioplasty were less likely to follow self-care behaviors compared with those undergoing open heart surgery. This is because of inadequate understanding of the severity of the disease, shorter hospitalization, and, faster recovery. The non-adherence to self-care behaviors gets worse over time [7]. These patients may tend towards self-care behaviors one month after the operation, but gradually, within 6 months their compliance is reduced significantly [6]. Thus, according to studies, 30–40% of patients undergoing angioplasty experience recurrent angina, Myocardial infarction, recurring angioplasty process, or death within a period of two years [8].

Although self-care behaviors are very important in these patients, the results of studies indicate that it is not well considered by patients [8–10]. In this regard, patient education plays an important role in empowering patients to actively participate in their care and better deal with their new condition [11]. The World Health Organization emphasized patient education as an important strategy to improve the active participation of patients in the management of their illness. Today, it is well established that the effectiveness of educational programs depends on the correct use of behavior change theories and appropriate educational techniques [12].

Considering the major role of knowledge, beliefs and interpersonal factors and generally the main modifiable factors known to somehow affect self-care in patients with cardiovascular diseases, the health promotion model proposed by Pender (1982) was used as the theoretical framework of the present study [13]. This model consisted of three concepts including individual characteristics and experiences, behavioral-specific cognition and effects, and behavioral outcomes. Prior related behavior and personal factors are included in the first concept. The concept of behavioral-specific cognition and effects included perceived barriers, perceived benefits, perceived self-efficacy, social support and norms, activity-related effects, and situation influences. Behavioral outcomes consisted of commitment to plan and conduct health-promoting behavior. Meanwhile, the prior related behavior, perceived benefits and barriers, self-efficacy, and social support are effective in explaining behavior in more than 50% of researches [14].

In the era of the internet and communication, the concept of education and the way people retrieve information has changed dramatically. In this regard e-learning and e-health have created many advantages in compression to traditional health services. Web-based education is one of the best forms of e-learning that addresses some of the limitations of traditional education and makes learning more accessible and flexible at any time and place to cover learners’ needs [15]. In the past few years, this type of education in behavior change interventions and web-based health-related educational programs to prevent and treat chronic diseases has led to interesting findings [16]. In general, considering the importance of the key role of self-care in cardiovascular patients undergoing angioplasty treatment after discharge from the hospital and considering that studies have shown that self-care in these patients is important to prevent the recurrence of the disease and its complications, it seems that the implementation of educational intervention based on the most important psychological structures affecting these behaviors can be effective. Therefore, considering the most important factors affecting the self-care of cardiovascular patients, Pender’s health promotion model was chosen as the theoretical framework of the study. On the other hand, taking into account the high potential of web-based education, which has been used in limited studies to improve self-care in chronic patients, the present study aimed to determine the effectiveness of web-based intervention based on the Pender’s health promotion model on self-care behaviors in patients with cardiovascular diseases under angioplasty.

## Methods

### Study design and population

This study is a clinical trial study. The research population consisted all patients undergoing angioplasty referring to Bushehr Heart Center.

### Sample size and sampling procedure

According to a study conducted by Westlake et al. [17], the mean score of physical activity as a self-care behavior in the web-based intervention group after three months was 40.1 ± 10.4 and it was 33.9 ± 9.3 in the control group. With a standard error of 5% and a power of 80%, the minimum required sample size was estimated at 40 for each group, using the formula:

1 $${n_1}={n_2}=\frac{{{{({z_{1 - \frac{\alpha }{2}}}+{z_{1 - \beta }})}^{^{2}}}(S_{1}^{2}+S_{2}^{2})}}{{{{\left( {{\mu _1} - {\mu _2}} \right)}^2}}}$$

Finally, with an approximate attrition rate of 20%, the sample size for each group was estimated at 50. Thus, the total sample size was 100.

Entering criteria in this study was: Elementary education, aged over 18 and under 70 years, speaking Persian, being treated by coronary angioplasty for the first time (because of: angina, acute coronary artery disease, myocardial infarction), lack of cognitive problems based on medical records, lack of participation in other self-care programs, having the ability to perform their daily activities, lack of advice on a specific diet, having left ventricular drainage fraction 40%≤, no need for open-heart surgery, patient or family member access to the smartphone, tablet, computer or smart TV, Internet access and willingness participate in the study. Exclusion criteria in this study were getting a new disease in a way that affects the ability of the subject to perform self-care and not watching the web-based educational content in the specified time.

For the sampling, at first the required permission was gained from the vice president of research at university. Arrangements were made with the hospital officials to do the sampling procedure. Considering the inclusion criteria, the final sample was selected among the patients undergoing angioplasty treatment in the post-angiography and CCU. The sampling continued until the desired sample size (*n* = 100) was met.

### Study instrument

The data gathering tool in this study consisted of three sections (demographic information, health promotion model constructs, and self-care behaviors).

Demographic information was measured using a form including 15 items about age, sex, marital status, education level, occupation, household income, co-occurrence of other diseases, history of heart disease in the family, drug use, smoking status, residential type, insurance coverage and type of insurance.

Constructs of health promotion model were evaluated using a researcher-made questionnaire prepared by a library survey on questionnaires of similar studies based on the Health Promotion Model. The questionnaire consisted of 59 questions with a 1 to 5 Likert scale about self-care behaviors that included perceived benefits (9 items), perceived barriers (21 items), perceived self-efficacy (17 items), and perceived social support (12 items). The reliability of the tool was calculated by Cronbach’s alpha coefficient for perceived benefit, perceived barriers, self-efficacy, and perceived social support 0.71,0.81,0.84, and 0.9 respectively.

The self-care behavior questionnaire consisted of 22 questions with five domains as follows: Dietary regimen (6 items), physical activity (6 items), adherence to treatment (6 items), smoking (3 items), and stress and anxiety control (2 items). The answers to these questions were based on a 5-point Likert scale (never, rarely, sometimes, most often, and always), with scores ranging from 0 to 4 and completed as self-reports. CVI and CVR for this tool were 0.94 and 0.87 respectively. The reliability of the tool was calculated by Cronbach’s alpha coefficient (0.72).

### Intervention

The educational package was prepared and then uploaded on the website and the participants were asked to view the course content completely within a period of 2 weeks. To guide the patients and their companions on how to access the website and receive the designed educational package, practical and face-to-face explanations were provided for 15–30 min at the patients’ bedside. The training for companions was also held by the researcher in the training class of the educational-therapeutic center. In the cardiac center of hospital through a briefing session, a pamphlet was provided on how to use the website and the participants were introduced to the training course. They were given a subscription card too, which contained the user ID, password, website address, and researcher’s phone number. The control group received only the routine hospital care. To prevent any exchange of information between the two groups, a card was provided to the patients which contained the username and password to enter the website of the intervention group upon discharge. Each patient was hospitalized in his own unit and had no connection with other patients.

After entering the website, the intervention group encountered a welcome message, then patients could complete their profile information, including age, gender, and other demographic characteristics, and even change their given password. Multimedia educational content was readily accessible on the user’s page. This multimedia patient education course consists of 8 sections all in plain language. The first part (introduction) includes information on the mechanism of the circulatory system, the risk factors for cardiovascular disease and the way coronary angioplasty is performed, necessary recommendations after discharge, and an introduction to the importance of self-care. The second section focuses on medication adherence in 7 separate episodes. The third section of the course focuses on dietary plans and healthy eating after discharge in 5 episodes. 4th section focuses on physical and sexual activity after discharge in 7 separate episodes. In the next section patients had access to a stress management video guide and in the 6th section the importance and strategies to quit smoking. 7th section of the intervention’s content has been specially developed for a family member or close friend of the patient to address the social support construct of the HPM. In this section, some practical recommendations for better care and support have been educated. Finally, in the 8th section, a role model who was treated with coronary angioplasty less than a year ago and had a tremendous good health status by self-care spoke in front of the camera and shared his experiences with the intervention group of patients.

During the educational intervention, the researcher was able to monitor the status of the course observation by the intervention group from the website management panel. After the end of the first week, the researcher contacted people who had not yet come to the site and encouraged them to view the course. The researcher also made sure during the phone call by asking questions about the content of the different parts of the course. In case of any problems in logging in to the website, patients would be contacted using the given support number. Then, two weeks after the scheduled deadline for viewing the course, all those who had fully viewed the educational content were invited to complete the questionnaires and those who had not yet viewed the educational content were given a week extra time. If someone did not receive web-based training at this time, would be excluded. Finally, two weeks and then three months after the web-based educational intervention, questionnaires were completed by the subjects in both the intervention and control groups. After the final assessment, in order to comply with ethics in the research, the participants in the control group were given a card containing a username and password to view the educational content along with a pamphlet on how to use the website.

### Data analysis

The collected data were analyzed by SPSS software version 22. In addition to the descriptive statistics, chi-square tests were used to compare the distribution of qualitative demographic variables; demographic characteristics (sex, education, marital status, income status, co-occurrence of other diseases) between the two groups before the intervention, and Independent T-test for comparing mean scores of specific emotions and cognitions of behavior (perceived benefits, perceived barriers, perceived self-efficacy, and social support) on adherence to self-care behaviors as well as self-care behaviors score in patients before and after the intervention. Repeated Measure ANOVA was used for evaluating and comparing the changes in the mean score of perceived benefits, perceived barriers, perceived self-efficacy, social support, and self-care behaviors in cardiovascular patients treated during angioplasty during the study and between two groups.

### Ethical considerations

The present research project was approved by the ethics committee of research at Bushehr University of Medical Sciences (#IR.BPUMS.REC.1395.56). Before the intervention and after providing full explanations to the patients and removing any ambiguities, the informed consent form for participation in the study was signed by the patients. During the study, inclusion in or exclusion from this study was voluntary for all patients. Also, the subscription of the participants to the website required a username and password that could be edited by the user and was anonymous.

## Results

### Demographic information

Participants in both intervention and control groups were homogeneous in all demographic characteristics except age, with no significant difference (Table 1). The mean age in the intervention group was 51.04 ± 7.73 and in the control group was 55.52 ± 7.78. The age group was not the same (*p* = 0.005).

**Table 1:** Distribution of demographic variables in research groups

		Intervention (*N* = 50)	Control (*N* = 50)	*P*-value
**Variables**		N (%)	N(%)	
Sex				
Male		37(%74)	28(%56)	0.093
Female		13(%26)	22(%44)	
Educational level				
Elementary		12(%24)	15(%30)	0.465
Highschool		28(%56)	31(%62)	
Academic		10(%20)	4(%8)	
Occupation				
Housekeeper		7(%14)	17(%34)	0.083
Employee		8(%16)	5(%10)	
Self-employed		22(%44)	14(%28)	
Retired		13(%26)	14(%28)	
History of disease				
Diabetes	Yes	16(%32)	16(%32)	0.099
	No	34(%68)	68(%68)	
Hypertension	Yes	11(%22)	17(%34)	0.265
	No	39(%39)	33(%66)	
Hyperlipidemia	Yes	13(%26)	12(%24)	0.099
	No	37(%74)	38(%76)	
Kidney disorders	Yes	3(%6)	4(%8)	0.099
	No	47(%94)	46(%92)	
Thyroid disorders	Yes	4(%8)	3(%6)	0.099
	No	46(%92)	47(%94)	
Monthly Income				
< 5000.000 IRR*		3(%6)	5(%10)	0.604
5 m to 10 m IRR		7(%14)	9(%18)	
10 m to 15 m IRR		19(%38)	21(%42)	
> 15.000.000 IRR		21(%42)	15(%30)	
Smoking				
Yes		15(%30)	18(%36)	0.671
No		35(%70)	32(%64)	

Among 100 participants who agreed to participate in the study, during the time of study,1 (2%) dropped out of the control group and 50 remained in the intervention group until the end of study.

Before the intervention, the two groups were similar in mean scores of health promotion model constructs (perceived benefits, barriers, self-efficacy, and social support). Repeated Measure ANOVA analysis showed that the intervention group had a significant difference in mean scores of perceived benefits, barriers, self-efficacy, and social support at different times during the study period (pre-intervention, two weeks, and three months after intervention). Comparing the changes between the two groups, the results showed that there was a significant difference between the intervention and control groups during the study, and according to Table 2, the results indicate that the changes in the mean score of the constructs in the intervention group were further increased compared to the control group (Table 2).

**Table 2:** Comparison of mean scores of health promotion model constructs during the study period in the intervention and control groups

Constructs	Time	Intervention group (*N* = 50) M ± SD	Control group (*N* = 49) M ± SD	*P*-value (between-group)
Perceived benefits	Before education	3.98 ± 0.39	3.83 ± 0.43	< 0.001
	2 weeks later	4.65 ± 0.30	3.1 ± 0.43	
	3 month later	4.97 ± 0.66	3.89 ± 0.41	
*P*-value (within group)				
Perceived barriers	Before education	2.60 ± 0.40	2.74 ± 0.33	< 0.001
	2 weeks later	1.69 ± 0.38	2.46 ± 0.31	
	3 month later	1.41 ± 0.32	2.49 ± 0.37	
*P*-value (within group)				
Perceived social support	Before education	1.62 ± 0.35	1.63 ± 0.43	< 0.001
	2 weeks later	2.70 ± 0.50	1.83 ± 0.44	
	3 month later	2.96 ± 0.47	1.70 ± 0.37	
*P*-value (within group)				
Perceived self-efficacy	Before education	2.71 ± 0.48	2.59 ± 0.45	< 0.001
	2 weeks later	4.15 ± 0.41	3.26 ± 0.44	
	3 month later	4.51 ± 0.31	3.28 ± 0.38	
*P*-value (within group)				

There was no statistically significant difference between the two groups in the mean scores of self-care behaviors before the intervention. After the educational intervention, the mean score of self-care behaviors (physical activity, diet, medication adherence, stress management, and anxiety control) was significantly increased in the intervention group. In the control group, the mean score of self-care behaviors also changed significantly during the study period. Comparing the differences between the two groups, the results showed that there was a significant difference between the intervention and control groups during the study (*P* < 0.001) except for controlling the smoking behavior (*P* = 0.380). The intervention group performed better on self-care behaviors (Table 3).

**Table 3:** Comparison of mean scores of self-care behaviors during the study period in the intervention and control groups

Self-care behaviors	Time	Intervention group M ± SD	Control group M ± SD	*P*-value (between-group)
Physical activity	Before education	1.24 ± 0.35	1.12 ± 0.40	< 0.001
	2 weeks later	2.22 ± 0.42	1.28 ± 0.39	
	3 month later	2.88 ± 0.46	1.39 ± 0.37	
*P*-value (within group)				
Healthy diet	Before education	2.40 ± 0.49	2.37 ± 0.42	< 0.001
	2 weeks later	3.66 ± 0.22	3.09 ± 0.37	
	3 month later	3.75 ± 0.20	3.25 ± 0.28	
*P*-value (within group)				
Medication adherence	Before education	1.69 ± 0.57	1.71 ± 0.42	< 0.001
	2 weeks later	3.36 ± 0.28	2.32 ± 0.41	
	3 month later	3.63 ± 0.28	2.37 ± 0.46	
*P*-value (within group)				
Stress management	Before education	0.085 ± 0.58	0.069 ± 0.62	< 0.001
	2 weeks later	2.36 ± 0.58	1.15 ± 0.55	
	3 month later	2.89 ± 0.58	1.25 ± 0.57	
*P*-value (within group)				
Non-smoking	Before education	2.53 ± 1.32	2.16 ± 1.32	0.380
	2 weeks later	3.47 ± 0.68	3.12 ± 0.95	
	3 month later	3.64 ± 0.73	3.08 ± 1.06	
*P*-value (within group)				
Self-care behaviors (Total)	Before education	1.71 ± 0.34	1.62 ± 0.24	< 0.001
	2 weeks later	3.05 ± 0.32	2.25 ± 0.37	
	3 month later	3.39 ± 0.29	2.34 ± 0.35	
*P*-value (within group)				

## Discussion

The present study aimed to investigate the effect of a web-based educational intervention on the self-care behavior of cardiovascular patients undergoing angioplasty based on Pender’s health promotion model.

The present findings showed that before the educational intervention, there was no statistically significant difference between the two groups in terms of perceived benefits. However, the training increased the patients’ perceived benefits of self-care behaviors in the intervention group. This finding is consistent with the results of studies conducted by Mohsenipouya et al. [18], Shujafard et al. [19], Lari et al. [20]. The results of a study by Lari et al. [20] on diabetic patients showed that the perceived benefits of physical activity had significant changes in these patients three months after the intervention. In another study by Mohsenipouya et al. [18], after the intervention, a significant increase was found in the perceived benefits score of performing self-care behaviors in coronary artery transplant patients. Our findings suggest that making changes in patient’s beliefs about the benefits of self-care behaviors is simple and achievable, and can even be accomplished using educational methods that require less cost and time.

The present findings showed that although there was no significant difference between the two groups in terms of perceived barriers before the educational intervention, the educational intervention reduced the barriers faced by patients in performing self-care behaviors in the intervention group. This finding was consistent with the results of studies conducted by Mohsenipouya et al. [18], Sharp et al. [21], Bakhshi et al. [22], Shojaeifar et al. [23], and Lari et al. [20]. The results of a study by Sharp et al.; [21] designed to determine the effectiveness of an educational intervention on self-efficacy and perceived barriers to healthy eating in cardiovascular patients over a period of 6 to 12 weeks after discharge showed that perceived barriers to healthy eating intake decreased significantly after the intervention. Shojaeifar et al. [23] in a study conducted among patients with hypertension found that there was a statistically significant difference between the mean of the main indicators of sensitivity, severity, benefits and barriers in the intervention and control groups before and after the intervention. These researchers managed to promote self-care behaviors in hypertensive patients. In general, web-based education seems to be an appropriate way of removing the mental barriers of patients with the dynamic and accessible features of any time and any place.

The present study showed no significant difference between the intervention and control groups before the educational intervention in terms of perceived self-efficacy. However, after the training, the patients’ self-efficacy to perform self-care behaviors increased significantly in the intervention group. This finding was consistent with the results of studies conducted by Baljani et al. [24], Mohsenipouya et al. [18], Wang et al. [25], Benitez et al. [26] and Kavita et al. [27].

Individuals’ perception of their ability to perform self-care called self-efficacy is an important determinant of self-care behaviors. Studies have shown that self-efficacy is a very effective factor for optimal self-care in cardiovascular patients as well as patients undergoing angioplasty [24]. According to Rees et al. [28], self-efficacy is the strongest predictor of self-care and has been consistently associated with acceptance of self-care behaviors such as diet modification, exercise, and nonsmoking in angioplasty patients. In this study, using self-efficacy-enhancing strategies such as enactive mastery, vicarious experience, providing positive feedback and verbal persuasion increased self-efficacy in patients. n the study of Wang et al. [25], which aimed to investigate the effect of multimedia educational programs on self-efficacy and physical activity in patients undergoing cardiac surgery, the results showed effective improvement in self-efficacy and physical activity of the patients up to one month after discharge. In the study of Benitez et al. [26], psychological construct-based intervention using web-based technology resulted in a significant increase one month after educational intervention in the self-efficacy of American adult participants. Since self-efficacy is a strong predictor of patients’ behavior, and determines the feeling, thought, and motivation of individuals, web-based education seems to have the potential to increase self-efficacy for better self-care.

The results of this study concerning the patients’ perceived social support and the effect of training showed that before the educational intervention there was no significant difference between the two groups. Yet, after the training, the patients’ perceived social support in performing self-care behaviors increased significantly in the intervention group. This finding is consistent with the results of studies conducted by Wilski et al. [29], Aggarwal et al. [30], Masoudnia et al. [31], Barrera et al. [32]. However, this finding is inconsistent with the study by Verheijden et al. [33]. Masoudnia et al. [31] found that coronary artery patients who felt they received more social support from friends, family members, and significant others adhered more to diet and medication after a bypass surgery. Barrera et al. [32] in a study using web-based education, were able to increase perceived social support in the self-management of diabetic patients. However the results of Verheijden et al.‘s [33] study among patients at the risk of developing cardiovascular diseases in Canadian families showed that after 4 and 8 months of follow-up, only 33 (24.73%) of patients in the intervention used the web. There was no statistically significant difference between the intervention and control groups in terms of changes in social support, which is not in line with the present study. The reason for this inconsistency can be attributed to the cultural behaviors of Iranian families regarding support and giving care to a patient member of the family. Overall, the results of the study suggest that designing web-based educational programs based on psychological constructs with emphasis on the supporting role of the family can influence the promotion of self-care behaviors in patients.

This study showed that changes in perceived benefits, barriers, social support and self-efficacy in patients can significantly improve self-care in patients, and despite these changes in the control group, the changes in the intervention group were significantly more after the intervention. This finding is in agreement with a body of research conducted by Mohsenipouya et al. [18], Lloyd et al. [34], Reid et al. [35], Ramadas et al. [36], Schweier et al. [37]. In a study by Lloyd et al. [34], a web-based intervention to improve self-care in heart failure patients after discharge in the United States within 30 days enhanced patients’ adherence to prescription medication by 75% and weight management by 84%. In a study by Schweier et al. [37] a three-month follow-up of a web-based intervention showed improvement in regular physical activity on a daily basis and the use of less fat for baking in patient. All of these studies confirm the results of this study, but since the constructs of the health promotion model were used in this study to change patients’ beliefs, it is possible to sustain self-care behaviors for a longer period. However, that approval would require further studies with a longer follow-up period.

Finally, based on the findings of the present study, it can be concluded that the use of psychological constructs based on health promotion model and providing information available to patients at any time through web-based education can improve self-care in patients undergoing angioplasty. In the present study, available routine patient education in the hospital was also able to significantly improve self-care behaviors in the control group, the results of this study showed that web-based training was more effective in promoting psychological constructs as well as improving self-care behaviors compared to the control group. In all cases, the differences between the intervention and control groups were statistically significant. On the other hand, patients who are undergoing angioplasty are more likely to visit their physician shortly after discharge and become more sensitive to the issue because of the newness of their problem, thus making self-care behaviors more and better. However, these patients usually do not have good self-care behaviors in the long run, and this further emphasizes the importance of training such as web-based training that can always be available to patients and has the potential for long-term effectiveness.

### Study limitations

There are a number of limitations in the present study as pinpointed here. Since most of the research questionnaire items were self-reported, there was always the possibility of misrepresentation or non-disclosure of information that sought to address this limitation by explaining the purpose of the study, emphasizing the confidentiality of the data, and encouraging them to provide accurate and truthful information. Failure to observe some parts of the curriculum was exemplified by the samples, explaining the importance of the course and encouraging them to continue to participate in the program. Future researchers are suggested to conduct web-based training with other electronic training, including applications and social networks, and cross-compare the findings. It is also suggested to perform this intervention with a similar approach yet with longer follow-ups or in other chronic diseases.

## Conclusion

Based on the results of the study, it seems that web-based education based on Health Promotion Model has a positive effect on changing patients’ beliefs about self-care behaviors and improving adherence to self-care behaviors in patients undergoing angioplasty procedure. Therefore, it is recommended to use web-based education as an adjunct to patient education programs to improve patients’ beliefs and self-care.

## Data Availability

The datasets generated and analyzed during the current study are not publicly available due to confidentiality and privacy related issues but are available from the corresponding author on reasonable request.
